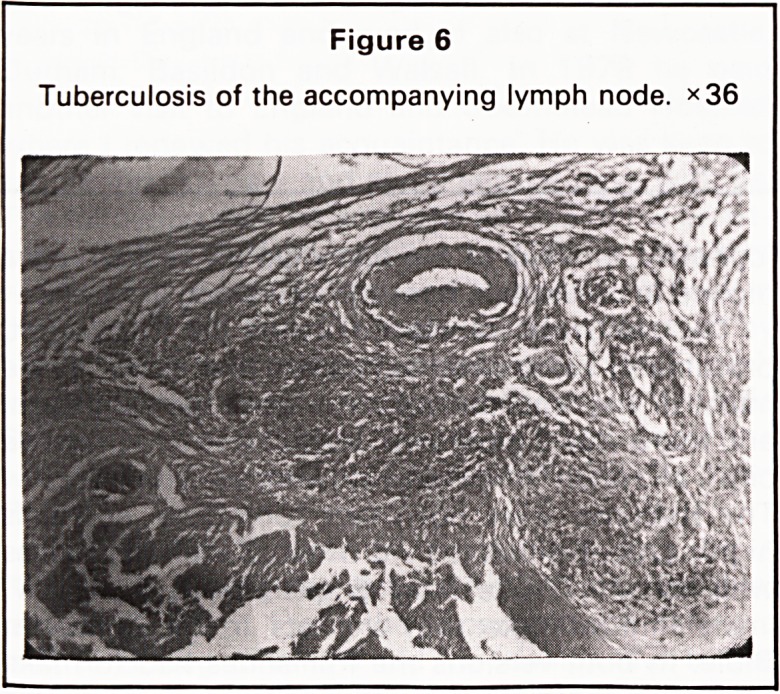# Gastric Tuberculosis

**Published:** 1983-04

**Authors:** M. M. Guirguis, Adly F. Ghaly, L. Abadir

**Affiliations:** SOHAG Teaching Hospital and Pathology Department, Ain Shams Faculty of Medicine; SOHAG Teaching Hospital and Pathology Department, Ain Shams Faculty of Medicine; SOHAG Teaching Hospital and Pathology Department, Ain Shams Faculty of Medicine


### Gastric Tuberculosis

**M. M. Guirguis F.R.C.S. (Ed. & Eng.), Adly F. Ghaly, M.D., Path. and L. Abadir, D.M.R.E.**
SOHAG Teaching Hospital and Pathology Department, Ain Shams Faculty of Medicine

### INTRODUCTION

Tuberculosis of the stomach is a rare disease. We were lucky enough to come across two cases. Good (1931) found three cases among 7410 consecutive gastric operations performed at the Mayo Clinic.

The incidence varies from 0.02 to 00.21% in routine autopsies and from 00.39 to 2.3% in autopsies on patients with pulmonary tuberculosis (Gaines et al., 1952).

Two cases were reported by Chatterjee and Dutt in 1955, one case by Baruah and Mahanta in 1958 and another case was reported by Amesur et al. in 1962. The striking features in all these four reported cases were the absence of tuberculous involvement of the lung together with the marked enlargement of the mesenteric lymph nodes.

Aird (1957) reported a co-existing tuberculosis ulcer of the stomach and the small intestine.

### CASE REPORTS

Both patients were females – aged 25 and 35 years respectively, anaemic, emaciated, and complaining of long standing epigastric discomfort with occasional vomiting.

### Case 1

The laboratory investigations showed the haemoglobin was 10 g/100 ml blood, the leucocytic count was 10,000/c.mm and sedimentation rate was 60 mm in the first hour. The urine and stool examination were normal.

A barium meal showed an irregular filling defect at the pylorus with an irregularity of the contour of the greater curvature at the pyloric antrum. The third part of the duodenum was completely obstructed at the site of crossing of the superior mesenteric vessels (Figures 1 and 2).

Laporatomy revealed a soft rounded mass about 2 inches in diameter in the pyloric canal. The left gastric, gastro-epiploic and subpyloric group of lymph nodes were enlarged. There was well-marked enlargement of the lymph nodes at the site of crossing of the superior mesenteric vessels over the third part of the duodenum, causing duodenal obstruction. the small intestine was normal but the caecum showed a soft mass with enlargement of the mesenteric, ilecolic and right colic lymph nodes.

**Figure 1**

Barium meal

Gastro-jejunostomy and ileo-transverse colostomy were carried out. Enlarged lymph nodes were taken for histology and proved to be caseating tuberculous lymphadenitis. The patient was put on long-term antituberculous treatment and made an uneventful recovery.

A chest X-ray was carried out in the immediate postoperative period and showed no abnormality.

The patient was checked at 6-months, she was symptom-free and her weight was increasing. A

**Figure 2**

Barium meal

**Figure 3**

Barium meal

**Figure 4**

Tuberculosis of the stomach. Very low power.

**Figure 5**

Tuberculosis of the stomach. ×36

barium enema was carried out 2 years later and showed the disappearance of the caecal lesion. However, the barium meal did not show any change in radiological appearance of the pyloric region.

### Case 2

The laboratory investigations showed the haemoglobin to be 9 g/100 ml. The sedimentation rate was 40 mm in the first hour, and the total leucocytic count was 7500/c.mm. The stool and urine examinations were normal.

A barium meal showed an irregularity of the greater curvature of the pyloric antrum with a small niche and irregular contour of the duodenal cap (Figure 3).

Laparotomy showed thickening and oedema of the pylorus and the first part of the duodenum. The lymph nodes along the greater and lesser curvature – especially those in the subpyloric group – were enlarged. The small and large intestine were normal but the mesenteric lymph nodes were enlarged and caseous. An anti-colic iso-peristaltic Polya Hoffmeister partial gastrectomy was carried out.

The pathological examination showed a rounded serpiginous ulcer of about 2 inches in diameter with undermined edges in the pyloric canal (Figure 4).

Microscopically, the section examined from both the ulcer and the lymph nodes showed a caseating tuberculous reaction (Figures 5 and 6). The patient was put on antituberculous treatment.

A chest X-ray taken in the immediate postoperative period showed no abnormality. Her postoperative period was uneventful.

**Figure 6**

Tuberculosis of the accompanying lymph node. ×36

### DISCUSSION

Gastric tuberculosis is very rare, inspite of the high incidence of gastric complaint in association with pulmonary tuberculosis. In gastric tuberculosis Morson and Dawson (1972) stated that it is unwise to diagnose the stomach lesion as being of tuberculous origin unless there is an open pulmonary tuberculosis. Contrary to this statement our two cases as well as the four cases reported in the papers quoted above were not associated with pulmonary tuberculosis.

As for the route of infection, Broders (1917) postulated four routes of infection. He considered the haematogenous route to be the most probable, due to the submucous site of the lesion. He considered direct infection from swallowed sputum to be debatable. He ascribed this to the bactericidal property of the gastric HCl, intact gastric mucosa, scarcity of lymph follicles in the wall of the stomach, relatively rapid emptying time of the stomach and possibly some inherent gastric resistance. He considered direct extension from neighbouring organs and retrograde lymphatic extensions to be rare. Contrary to this statement in our two cases and the four reviewed cases, there was marked enlargement and caseation of the mesenteric glands.

Lymph gland enlargement was a prominent feature. There is a direct communication between the lymph nodes around the root of the superior mesenteric vessels and the subpyloric group of lymph glands (Gray's anatomy, 1973). This favours our view that the most likely route of infection is through lymphatic spread from the mesenteric group of lymph glands and explains the frequency of involvement of the pylorus in gastric tuberculosis.

According to Morson and Dawson (1972) the characteristic lesion is a deep ulcer with little surrounding fibrosis. More rarely a large inflammatory mass of tuberculous granulation tissue is present. One of our cases was of the ulcerative type and the other was of the hypertrophic type.

The history, physical examination and laboratory findings offer little help in the diagnosis of gastric tuberculosis. Radiological findings may cast some light on the diagnosis, the presence of indentation of the greater curvature and involvement of the first part of the duodenum in a young subject is suspicious but in nearly every case, diagnosis is suspected at operation and confirmed by histopathology.

Vajo (1965) stressed the fact that medical treatment is of no use. Partial gastrectomy, if the general and local condition allows, is the treatment of choice, because this will remove the focus of infection. Gastrojejunostomy was carried out for the first case because of the bad general condition of the patient and the associated caecal lesion.

### SUMMARY

Two cases of gastric tuberculosis without pulmonary lesion were studied and reported, and four cases reported in the literature are reviewed. In all cases the pylorus was the site of involvement, and there was marked tuberculous involvement of the mesenteric and subpyloric groups of lymph glands.

In our view infection is due to direct lymphatic spread from the mesenteric to subpyloric group of glands rather than to haematogenous spread as postulated by Broders.

### REFERENCES

AIRD, I. (1957) Companion in surgical studies. Livingstone. London.

AMESUR, N. R., AMESUR, H. G. and CHABBRA, A. S. (1962) *J. Ind. Med. Assosn.* **38**, 548.

BARUAH, B. D. and MAHANTA J. (1958) *J. Ind. Med. Assosn.* **31**, 436–438.

BRODERS, A. C. (1917) *Surg. Gynaec. & Obst.* **25**, 490.

CHATTERJEE, S. K. and DUTT, A. K. (1955) *Ind. J. Surg.* **17**, 258.
GAINES, W., STEINBACH, H. L. and LOWENHAUPT, E. (1952) *Radiology* **58**, 808.
GOOD, R. W. (1931) *Arch. Surg.* **22**, 415.
GRAY'S ANATOMY (1973) Thirty-fifth edition. Longman. London.
MORSON, B. C. and DAWSON I. M.P. (1972) Gastro-intestinal pathology. Blackwell. London.
VAJO, E. (1956) *Minerva Chir.* **11**, 1200.

## Figures and Tables

**Figure 1 f1:**
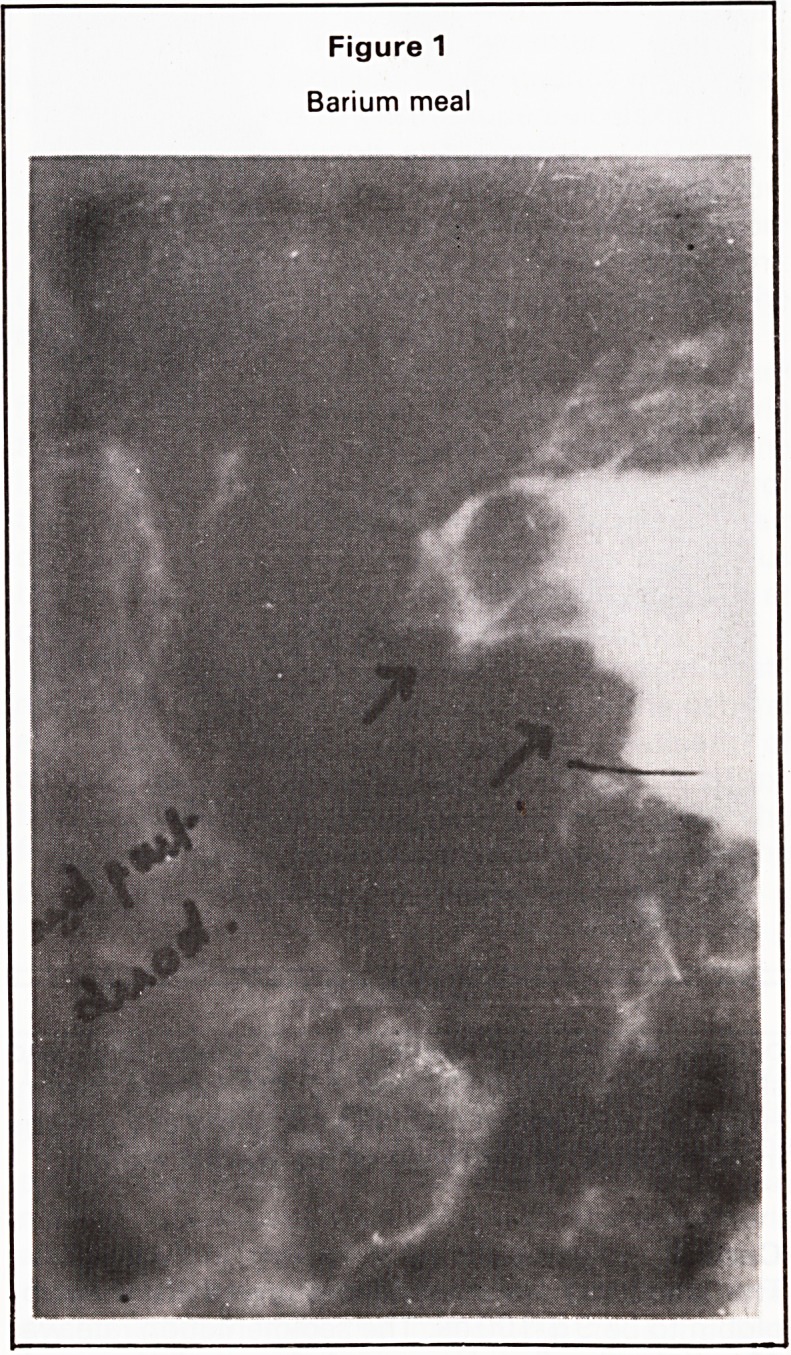


**Figure 2 f2:**
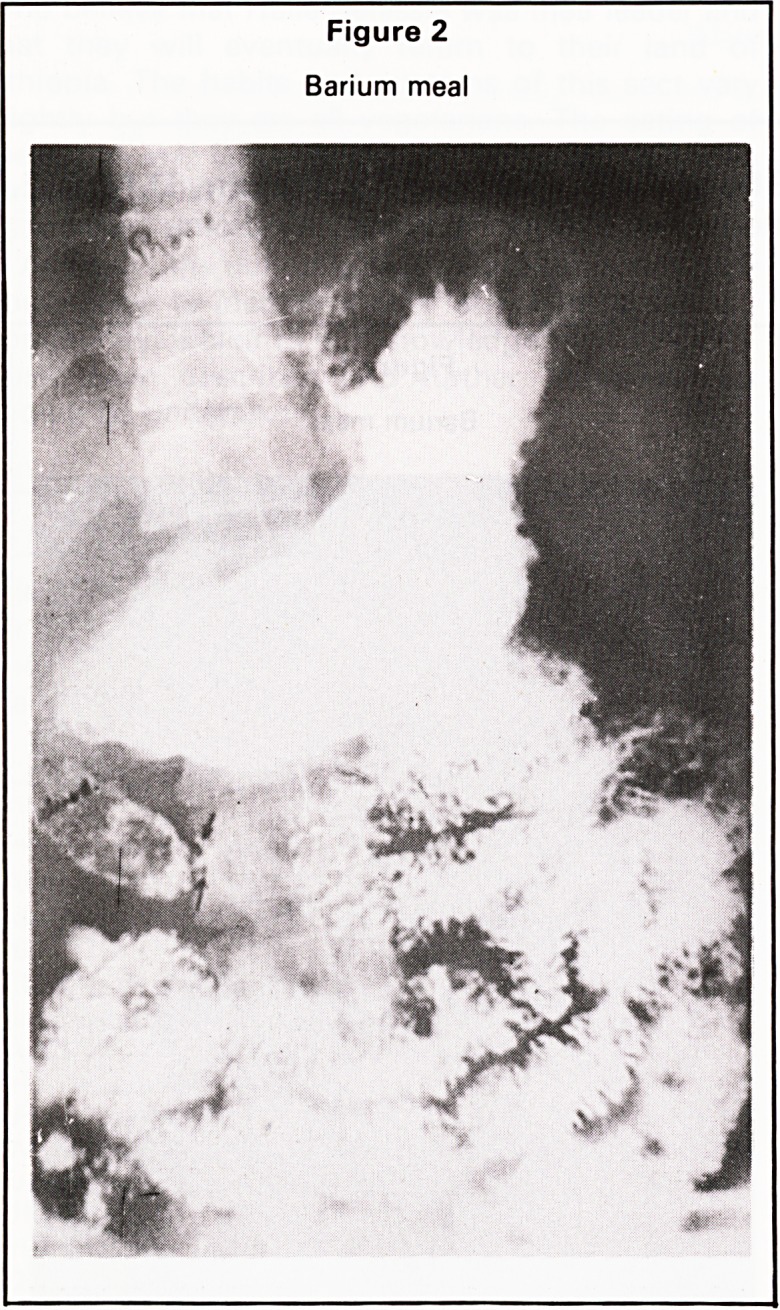


**Figure 3 f3:**
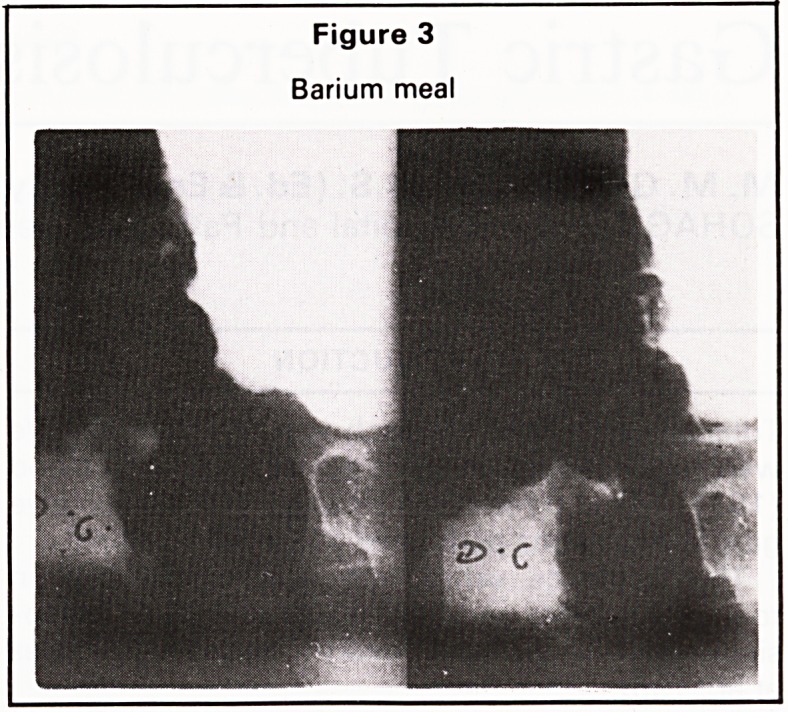


**Figure 4 f4:**
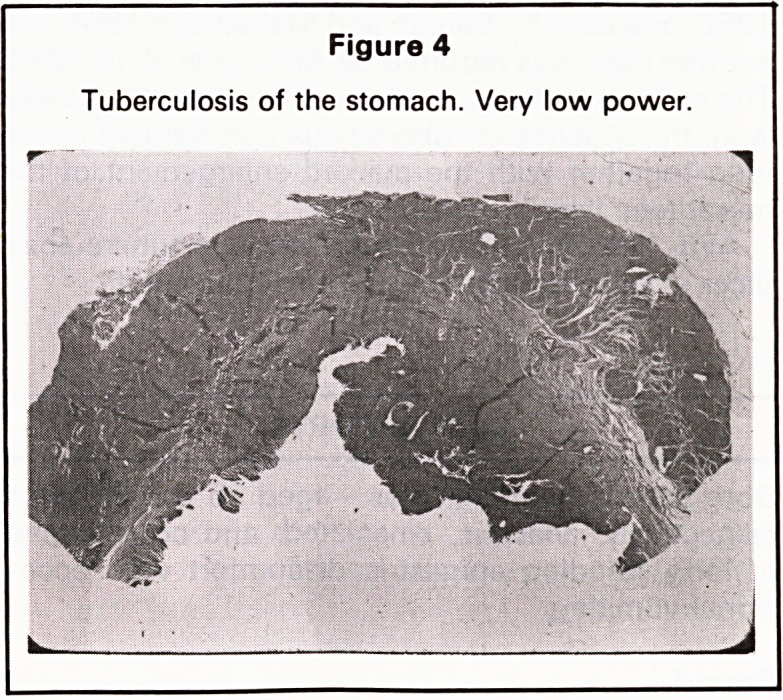


**Figure 5 f5:**
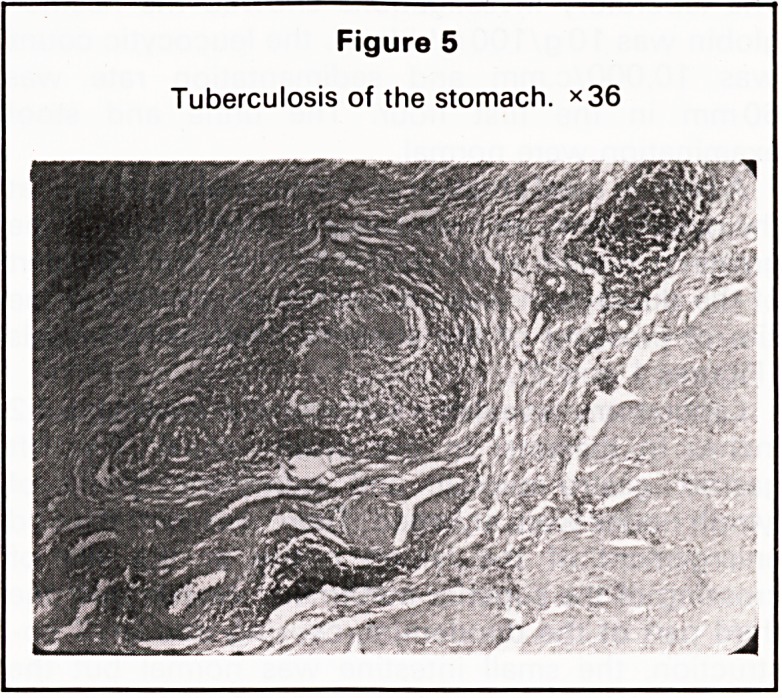


**Figure 6 f6:**